# Etiology and Outcome of Patients with Upper Gastrointestinal Bleeding: A Study from South of Iran

**DOI:** 10.4103/1319-3767.70608

**Published:** 2010-10

**Authors:** Mohammad J. Kaviani, Mohsen Pirastehfar, Ali Azari, Mehdi Saberifiroozi

**Affiliations:** Internal Medicine Department and Gastroenterohepatology Research Center, Shiraz University of Medical Sciences, Shiraz, Iran; 1 Gastroenterohepatology Research Center, Shiraz University of Medical Sciences, Shiraz, Iran

**Keywords:** Acute upper gastrointestinal bleeding, gastric ulcer, nonsteroidal antiinflammatory drugs

## Abstract

**Background/Aim:**

The prevalence of acute upper gastrointestinal bleeding (AUGIB) has undergone a change after implementation of eradication therapy for *Helicobacter pylori* in peptic ulcers effective prevention of esophageal variceal bleeding and eventually, progressive use of low dose aspirin and other nonsteroidal antiinflammatory drugs (NSAIDs). To evaluate this subject, we performed a prospective study in two main University Hospitals of Shiraz (the largest city of southern Iran).

**Materials and Methods:**

All adults who were admitted in emergency room with impression of AUGIB and existing patients who developed AUGIB were included in the study. Gastroscopy was done with a follow-up for the next 15 days.

**Results:**

572 patients (mean age: 54.9 years) entered in the study. The most common presenting symptom was hematemesis or coffee-ground vomits (68%). 75% of patients gave history of consumption of low dose aspirin or other NSAIDs regularly. Gastric and/or duodenal ulcers were the most common causes (252/572, 44%) of AUGIB (Gastric ulcer: 173/572, 30% and duodenal ulcer: 93/572, 16%, respectively). Esophageal varices were the third common cause (64/572, 11%). 36 (6%) of the patients died. Mean age of these patients was higher than the patients who were alive (64.8 vs. 54.2 years, *P* = 0.001). Other than age, orthostatic hypotension on arrival (267/536 vs. 24/36, *P* = 0.018) and consumption of steroids (43/536 vs. 10/36, *P* = 0.001) were significant factors for increasing mortality.

**Conclusions:**

The most common cause of AUGIB, secondary only to NSAIDs consumption, is gastric ulcer. Mortality of older patients, patients who consumed NSAIDs and steroids concomitantly, and patients with hemodynamic instability on arrival were higher.

Acute upper gastrointestinal bleeding (AUGIB) remains a common emergency and potentially fatal situation that requires hospitalization. The incidence of AUGIB varies between 50-150 hospital admissions per 100,000 population in a year[1–5] (approximately 1% of all emergency room admissions).

Approximately 45–60% of admissions for AUGIB worldwide are due to peptic ulcers followed by esophagitis and esophageal varices.[2,6] Although, *H. pylori* infection has been one of the most common causes of peptic ulcer disease, and eventually AUGIB, in the developing countries in the last few years, it seems that due to better sanitation, better diagnostic and therapeutic approaches, rate of AUGIB secondary to *H. pylori* infection has been decreased.[4,7,8] On the other hand, excessive usage of low dose aspirin for primary or secondary prevention of atherosclerotic heart and brain diseases, increasing life expectancy and so increasing rate of degenerative joint disease and osteoarthropathies and excessive ingestion of other nonsteroidal antiinflammatory drugs (NSAIDS), may change the incidence, age of presentation, site of bleeding and outcome of patients with nonvariceal AUGIB in the last decade.[7,8]

It seems that better sanitation, vaccination against Hepatitis B virus, prophylactic using of propranolol, esophageal band ligation and liver transplantation has changed the incidence of esophageal variceal bleeding.[9]

Common use of high dose proton pump inhibitors, better availability of diagnostic and therapeutic endoscopy and increasing cost of hospitalization may change the economic burden of AUGIB.[10]

Despite the fact that epidemiologic data are important to get insight into the actual situation,[11] there is no epidemiologic survey regarding AUGIB in our area.

The aim of this study was to survey the etiology and clinical outcome of AUGIB in referred and already hospitalized patients of two hospitals in Shiraz.

## MATERIALS AND METHODS

We prospectively evaluated clinical characteristics, cause of bleeding and clinical outcome, of 383 referred (*de novo*) and 189 already admitted (inpatients) referred to the Faghihi Hospital and Namazi Hospital.

### Patients

*De novo* patients: All adult patients (≥16 years old) who were admitted in emergency room with impression of AUGIB by internal medicine residents for more than 8 hours, were included in the study. All patients were admitted with a history of malaena or hematemesis on the day of admission

### Ongoing hematemesis/melena

This was defined as a history of melena/hematemesis several days before admission and decrease in the hemoglobin level (>1gm/dl), shock (blood pressure <90/60 mmHg in supine position), pallor, orthostatic hypotension (>20 mmHg decrease in systolic blood pressure or >10mmHg in diastolic blood pressure from supine to standing position), or anemia (hemoglobin < 12 gm/dl and <14 gm/dl in female and male respectively), insertion of naso-gastric tube and suction of fresh blood or coffee-ground materials without clearance of gastric washing by 250 cc of isotonic solution and exclusion of other causes of false AUGIB, such as bleeding from upper respiratory tract, nose bleeding, bleeding from paranasal sinuses, etc.

In-hospital patients: AUGIB in hospital adult (≥16 years old) patients were confirmed with nonclearance of gastric washing by 250 cc of isotonic solution, positive stool occult blood test and no evidence of active bleeding from upper respiratory tract.

After enrolment in the study, a questionnaire including demographic data, important points in the history, physical exam and laboratory tests such as history of acid peptic disease, presence of cirrhosis, cause of cirrhosis, NSAID use, regular ingestion of low dose aspirin, previous history of AUGIB and cause of it, co-morbidities, ongoing vital signs, pallor, organomegaly, ascites, ongoing and 6 hour after admission hemoglobin (Hgb), activated prothrombin time (PT), platelet (Plt), partial thromboplastin time (PTT) etc was filled by research assistants. Upper GI endoscopy was performed by our on-call fellow or attending physician within 24 hours of admission. There was a daily follow-up of patients after admission and up to 15 days after being discharged from hospital. End points including mortality, re-bleeding in hospital and within 15 days after discharge, blood transfusion and surgery were registered.

### Statistical analysis

Statistical package for social sciences (*SPSS, version* 15.0; Chicago, IL, USA) and Epi Info 2000 programs were used for data analysis. Student T-test was used for quantitative and Chi-square and Fisher exact tests were used for qualitative variables.

## RESULTS

Five hundred and seventy two patients (including 383 *de novo* and 189 in-hospital) entered the study, of which 377 (66%) were male. The mean age of our patients was 54.9 years (±SD: 18.7). Other demographic data are shown in Table 1.

**Table 1:** Comparison and common complaints of patients with upper gastrointestinal bleeding

	Whole No.: 572 (%)	*de novo* No.: 383 (%)	In-hospital No.: 189 (%)	*P* value (*de novo* vs. In-hospital)
Sex				
Male	377 (66)	257 (67)	120 (64)	NS#
Female	195 (34)	126 (33)	69 (36)	
Mean age/yr (±SD)	54.9(18.7)	55(18.8)	54.8(18.7)	NS
History related to bleeding				
Melena	217(38)	147 (38)	70 (37)	NS
Hematemesis or coffee-ground vomitus	390(68)	263 (69)	127 (67)	NS
Fainting or dizziness	77(14)	54(14)	23(12)	NS
Hematochezia	22(4)	15(4)	7(4)	NS
Previous history of				
Duodenal ulcer	24(4)	16(4)	8(4)	NS
Gastric ulcer	67(12)	49(13)	18(10)	NS
Portal hypertension	12(2)	6(2)	6(3)	NS
Cirrhosis	38 (7)	29 (8)	9(5)	NS
Gl bleeding	66 (12)	47 (12)	19(10)	NS
Co-morbidities	400 (70)	254 (66)	146 (72)	0.007
Ischemic heart disease	34(9)	20 (8)	14(10)	NS
Renal failure	9(2)	6(2)	3(2)	NS
Cerebro-vascular accident	37 (9)	28(11)	9(6)	NS
Malignancies	37 (9)	23 (9)	14(10)	NS
Hypertension	87 (22)	63 (25)	24(16)	0.05
Trauma	22 (6)	9(4)	13(9)	0.02
Others	174 (44)	105 (41)	69 (47)	NS
History of drug consumption				
ASA	172 (30)	118(31)	54 (29)	NS
Celecoxib	10(2)	6(2)	4(2)	NS
Other NSAIDs	255 (45)	166 (44)	89 (47)	NS
Steroids$	53 (9)	31 (8)	22 (12)	NS
Warfarin	42(7)	29 (8)	13(7)	NS
Alcohol consumption	48 (8)	34(9)	14(7)	NS
History of smoking or addiction				
Water-pipe	139 (24)	91(24)	48 (25)	NS
Cigarette	199 (35)	137 (36)	62 (33)	NS
Morphine derivatives	35 (6)	26 (7)	9(5)	NS

#: NS means statistically not significant

$: 45/53 of these patients consumed NSAIDs (16 of them, low dose aspirin and 25 others consumed other types of NSAIDs) concomitantly

The most common symptom on presentation was hematemesis or coffee-ground vomits (68%) followed by melena (38%) in our patients. Sixteen percent of our patients gave history of acid peptic disease and 75% of them consumed low dose aspirin or other NSAIDs (except for celecoxib) regularly. Other basic histories are shown in Table 1. On admission, 42% of our patients had orthostatic hypotension, with a mean hemoglobin level of 10.9 gm/ dl [Table 2].

**Table 2:** Clinical features on admission and primary laboratory results of patients with *de novo* upper gastrointestinal bleeding

Test	Result No.: 383 (%)
Orthostatic hypotension#	155 (42)
Shock$	8 (2)
Splenomegaly	28 (7)
Mean hemoglobin/gm/dl (±SD)	10.9 (2.5)
Abnormal prothrombin time	69 (18)
Abnormal partial thromboplastin time	67 (18)
Mean platelet/mm^3^ (±SD)	215227 (103790)
Mean creatinine/mg/dl (±SD)	1.8 (5.3)

#: Drop of 20 mmHg or 10 mmHg in systolic or diastolic blood pressure respectively after 3 minutes sitting

$: Blood pressure < 90/60 mmHg

Gastric ulcer was the most common finding in upper endoscopy. One hundred and seventy three patients had gastric ulcer (124/383, 32% and 49/189, 26% of *de novo* and in-hospital patients, respectively). The most common site was lesser curvature (57/173, 33%) and only 87/173 (50%) of these ulcers fulfilled criteria of low risk ulcer for rebleeding. Duodenal ulcer was present in 93 patient (62/383, 16%, and 31/189, 16% of *de novo* and in-hospital patients, respectively). 55/93 (59%) of these ulcers fulfilled criteria of low risk ulcer for re-bleeding. Esophageal varices were found in 64 patients (47/383, 12% and 17/189, 9% of *de novo* and in-hospital patients respectively). Eight of these patients had gastric ulcer (four Clean-based, one with oozing of blood from ulcer bed, and three with visible vessel) and five of them had clean based duodenal ulcer. Less than 10% of our patients had gastritis or Mallory-Weiss’ tears as the causes of AUGIB and 99/572 (17%) of our patients had normal upper endoscopy [Table 3].

**Table 3:** Result of primary endoscopy in *de novo* and in-hospital patients with upper gastrointestinal bleeding

	No.: 383 (%)	No.: 189 (%)
Abnormal findings in esophagus	117(31)	53 (28)
Varices#	47	17
GERD^$^	23	14
Other esophagitides	8	3
Mai lory-Weiss–s tears ± ulcer	34	13
Mass	5	6
Abnormal findings in stomach	150 (39)	58 (31)
Ulcer	124	49
Site of ulcer		
Cardia	9	3
Fundus	32	14
Lesser curvature	38	19
Greater curvature	22	3
Antrum	11	2
Pre-pyloric area and pylorus	12	8
Description of ulcer/s		
Clean based	41	22
With simple clot	19	5
With oozing of blood from ulcer bed	34	15
With adherent clot	13	3
With visible vessel	13	2
With spurting artery	4	0
Gastritis	16	2
Mass	6	4
Vascular ectasia	4	3
Abnormal findings in duodenum	63(16)	32 (17)
Ulcer	62	31
Description of ulcer/s		
Clean based	34	14
With simple clot	4	5
With oozing of Wood from ulcer bed	12	7
With adherent clot	4	3
With visible vessel	4	3
With spurting artery	4	1
Vascular ectasia	1	0
Mass	0	1
Normal	53 (14)	46 (24)

#: 13/64 of these patients had concomitant gastric and/or duodenal ulcer

Injection therapy and/or argon plasma coagulation was done for 54 patients with high risk ulcers and 12 (22%) of them had high risk ulcers in second look endoscopy. Blood transfusion was required in 197 patients and finally, mortality rate of our patients was 6% for *de novo* and 7% for in-hospital patients respectively [Table 4].

**Table 4:** End points in *de novo* and in-hospital patients with upper gastrointestinal bleeding

	Whole No.: 572	*de novo* No.: 383	In hospital No.: 189
Injection therapy ± APC of ulcer	54	42	12
Sclerotherapy of esophageal varices	53	40	13
Rubber band ligation	11	7	4
Blood transfusion	197	98	99
Re-endoscopy	76	39	37
High risk ulcers in re-endoscopy	12	10	2
Mean hospital staying /day (±SD)	3.7 (4.4)	2.4 (2.2)	6.3 (6.3)
Death in hospital due to acute UGI bleeding	36 (6%)	22 (6%)	14 (7%)

The mortality rate was higher in older patients, patients with orthostatic hypotension on arrival and patients who consumed steroids [Table 5].

**Table 5:** Comparison between alive and deceased patients with upper gastrointestinal bleeding

	Whole No.: 572 (%)	Alive No.: 536 (%)	Deceased No.: 36 (%)	*P* value (Alive *vs*. deceased)
Sex				
Male	377 (66)	351 (65)	26 (72)	NS#
Female	195 (34)	185 (35)	10(28)	
Mean age/ yr (±SD)	54-9(18.7)	54.2 (18.7)	64.8(16.3)	0.001
History related to bleeding				
Melena	217 (38)	200 (38)	17(47)	NS
Hematemesis or coffee-ground vomitus	390 (68)	367 (69)	23(64)	NS
Fainting or dizziness	77(14)	72 (13)	5(14)	NS
Hematochezia	22(4)	22(4)	0	NS
Previous history of				
Duodenal ulcer	24(4)	23 (4)	1(3)	NS
Gastric ulcer	67(12)	59(11)	8(22)	0.04
Portal hypertension	12(2)	12(2)	0(0)	NS
Cirrhosis	38 (7)	36 (7)	2(6)	NS
Gl bleeding	66(12)	61 (11)	5(14)	NS
Peptic ulcer	6	6	0	NS
Varices	49	44	5	NS
Co-morbidities	400 (70)	370 (69)	30 (83)	NS
Ischemic heart disease	34	32	2	NS
Renal failure	9	8	1	NS
Cerebro-vascular accident	37	36	1	NS
Malignancies	37	35	2	NS
Hypertension	87	80	7	NS
Trauma	22	19	3	NS
Others	174	160	14	NS
History of drug consumption:				
ASA	172 (30)	161 (30)	11 (31)	NS
Celecoxib	10(2)	10(2)	0	NS
Other NSAIDs	255 (45)	239 (45)	16(44)	NS
Steroids	53 (9)	43 (8)	10 (28)$	0.001
Warfarin	42 (7)	39 (7)	3(8)	NS
Mean second hemoglobin(gnVdl)	10.9	10.9	10.8	NS
Orthostatic hypotension on arrival	291 (51)	267 (50)	24 (67)	0.018
Abnormal prothrombin time	101 (18)	94 (18)	7(19)	NS
Partial thromboplastin time	104(18)	96 (18)	8(23)	NS
Endoscopic findings				
Esophageal varices	64(11)	59(11)	5(14)	NS
GERD	37(6)	35 (7)	2(6)	NS
Esophageal ulcer	47 (8)	44(8)	3(8)	NS
Esophageal mass	11(2)	10(2)	1(3)	NS
Gastric ulcer	173 (30)	163 (30)	10(28)	NS
Gastritis	18(3)	14(3)	4(11)	0.02
Mass	10(2)	8(1)	2(6)	NS
Duodenal ulcer	93(16)	89 (17)	4(11)	NS
Hospital staying/day in *de novo* patients	2.4	2.4	3.3	0.045

#: NS means statistically not significant

$: 7/10 of these patients consumed aspirin or other NSAIDs concomitantly

Analysis showed that mortality was directly related to increase in age [Table 6].

**Table 6:** The mortality rate and age of patients with upper gastrointestinal bleeding

Age group in years (mortality %)	Alive	Deceased
<50 (4)	206	8
50–59 (3)	97	3
60–69 (6)	104	7
70–79 (14)	80	13
80–89 (9)	50	5
≥90 (0)	2	0
≤60 (4)#	300	11
>60 (10)	236	25

#: Mortality rate between ≤60 years old patients and higher than 60 years old patients were significant (*P* value: 0.003)

Comparison between ≤60 and >60 years old patients showed that older patients had more serious presentations (hematemesis or coffee-ground vomits, orthostatic hypotension or shock on arrival). They consumed aspirin, alcohol, steroids, warfarin, smoked water-pipe and cigarettes more than younger patients and eventually, acid peptic disease as the cause of AUGIB was more frequent in older patients [Table 7]. Thirty four patients who consumed steroids in this age group, also consumed aspirin or other NSAIDs and seven of these patients died. Acid peptic disease as the cause/s of AUGIB was more frequently found in older patients [Table 7].

**Table 7:** Comparison between younger (≤60 years) and older (>60 years) patients with upper gastrointestinal bleeding

	≤60 years No: 311 (%)	>60 years No: 261 (%)	*P* value
Male/Female	205/106	172/89	NS#
*de novo*/In hospital	207/104	176/85	NS
Melena	127 (41)	90 (34)	NS
Hematemesis or coffee ground vomitus	200 (64)	190 (73)	0.019
Co-morbidities			
Ischemic heart disease	14(5)	20 (8)	
Cerebrovascular accident	5(2)	32(12)	
Hypertension	25 (8)	62 (24)	
Trauma	17(5)	5(2)	
NSAID consumption	191 (61)	246 (94)	0.0001
ASA	44 (14)	128 (49)	0.0001
Celecoxib	5(2)	5(2)	NS
Other NSAIDs	142 (46)	113(43)	NS
Steroid consumption	13(4)	40(15)$	0.0001
Warfarin consumption	11 (4)	31 (12)	0.0001
Alcohol consumption	19(6)	29 (11)	0.035
Water pipe smoking	49 (16)	90 (34)	0.0001
Cigarrette smoking	92 (30)	107 (41)	0.005
Orthostatic hypotension on arrival	146 (48)	145 (57)	0.027
Shock on arrival	8(3)	18(7)	0.015
Major causes of bleeding			
Esophageal varices	36 (12)	28 (11)	NS
GERD	25 (8)	12(5)	NS
Mallory-Weiss’ tear/ulcer	22 (7)	25(10)	NS
Esophageal mass	4(1)	7(3)	NS
Gastric ulcer	86 (28)	86 (33)	0.03
Gastritis	12(4)	6(2)	NS
Gastric mass	6(2)	4(2)	NS
Duodenal ulcer	44 (14)	49(19)	0.04
Gastric ulcer and/or duodenal ulcer	124 (40)	128 (49)	0.028

#: NS: Statistically not significant

$: 34/40 of these patients consumed aspirin (13 patients) or other NSAIDs (23 patients) concomitantly and 7/10 of patients who concomitantly consumed aspirin (2/10) or other NSAIDs (5/10) and steroids deceased

## DISCUSSION

Because of shortcomings in records, this study was done prospectively with predefined strategy and goals in the two largest referral centers in southern Iran i.e., Faghihi and Namazi Hospitals.

Only patients who were admitted in hospital for a minimum of 8 hours were considered for the study and followed up to the end point, so automatically patients with minor bleeding were excluded. As a result, in our study the most common presentation of AUGIB was hematemesis or vomiting of coffee-ground material in both *de novo* and in-hospital patients [Table 1]; orthostatic hypotension was seen in 291 (51%) of patients, the most common cause of AUGIB was acid peptic disease and not gastritis (such as study of Boonpongmanee S. *et al*[5]), and only 63/173 (36%) of gastric ulcers and 46/93 (49%) of duodenal ulcers were clean-based.

As a developing country, statistical analysis of demographic data in this survey showed that there is no difference between our results in sex (66% male) and mean age (54.9 year old) in comparison with the same studies in developed countries.[2,4]

Due to progressive and drastic changes in medical and health care systems, the age and life expectancy of general population has been increasing in Iran, and in this way some of the common geriatric problems such as coronary artery disease, degenerative joint disease and osteoarthropathies and eventually excessive consumption of all kinds of NSAIDs and low dose aspirin as main standard therapies has increased. It seems that this expansive usage of these drugs led to increasing rate of GU as a cause of AUGIB and this may be comparable with results of Enestvedt *et al*.[1] On the other hand, according to the above facts and considering the effective newer and widespread management of *H. pylori* as a major cause of peptic ulcer and gastric cancer, introducing gastric ulcer as a new leading cause of AUGIB is confirmed by our study (GU 30%, DU 16%).[10,12] Meanwhile, the decreasing rate of variceal bleeding (11%) may be explained by the extensive usage of effective drugs for chronic hepatitis/cirrhosis caused by hepatitis B and C viruses and autoimmune hepatitis, in addition to a vast usage of propanolol and rubber band ligation as primary prevention methods for variceal bleeding.

Comparing similar studies,[13] the reduction in hospital stay, from a standard 5.5 days to 2.2 days, can be explained by better management of AUGIB.

Mortality rate of AUGIB in our centers was comparable to centers in developed countries.[11] We compared some of the data between alive and deceased patients.

The first finding was mean age (54 vs. 65 years, *P* = 0.001). Mortality rate was significantly different for. 60 years (4%) versus >60 years (10%, *P* = 0.003). Further analysis showed that this finding may be secondary to more blood loss before referral [Table 7] and frequent consumption of aspirin or other anticoagulants.[3,14] Further, this is also secondary to concomitant diseases such as ischemic heart disease and cerebro-vascular accident, which have a higher frequency in older patients. Acid peptic disease as the major cause of AUGIB was more frequent in older patients (40% in younger vs 49% in older patients respectively, *P* = 0.028). The second finding in deceased patients was past history of steroids usage which also was statistically different (8% in alive Vs 28% in deceased patients respectively, *P* = 0.001). External steroids, by themselves, are not harmful for stomach or duodenum but concomitant use of steroids and NSAIDs usually increase the risk of gastric and duodenal ulceration and AUGIB.[15] Our study showed (just like Shorr *et al* findings)[16] that concomitant use of steroids and NSAIDs may also increase the mortality secondary to AUGIB in this subset of patients. 45 out of 53 (85%) steroids users concomitantly used NSAIDs, while 7 out of 10 died due to AUGIB.

Acute (15-day) mortality rate in patients with esophageal variceal bleeding (5/64, 8%) was comparable with other nonvariceal causes such as acid peptic disease (13/252, 5%) and this may be secondary to better management of these patients and/or use of newer therapeutic modalities to stop the bleeding in this group. This result agrees with findings of Chalasani N *et al*.[9]

In conclusion, it appears that the most common cause of AUGIB is acid peptic disease, which increases with the age of the patient, and frequent consumption of NSAIDs, and gastric ulcer as the main source of bleeding may be NSAIDs induced. It is therefore suggested to take a thorough history pertaining to acid peptic disease, before the start of aspirin, and decrease the threshold of performing upper gastrointestinal endoscopy and possibly prescription of proton pump inhibitors especially in high risk groups (older age, concurrent anticoagulant, or steroid users, etc). Furthermore eradication of *H. pylori* prior to starting aspirin or other NSAIDs may decrease the rate of AUGIB secondary to acid peptic disease.[11] Finally, in this regard, consideration of international recommendations on starting aspirin or other NSAIDs will be helpful.
